# The Good, the Bad and the Unknown of CD38 in the Metabolic Microenvironment and Immune Cell Functionality of Solid Tumors

**DOI:** 10.3390/cells9010052

**Published:** 2019-12-24

**Authors:** Jessica M. Konen, Jared J. Fradette, Don L. Gibbons

**Affiliations:** 1 Department of Thoracic/Head and Neck Medical Oncology, University of Texas MD Anderson Cancer Center, 1515 Holcombe Blvd, Houston, TX 77030, USA; jmkonen@mdanderson.org (J.M.K.); jjfradette@mdanderson.org (J.J.F.); 2 Department of Molecular and Cellular Oncology, University of Texas MD Anderson Cancer Center, 1515 Holcombe Blvd, Houston, TX 77030, USA

**Keywords:** immune suppression, ectoenzymes for NAD and ATP metabolisms, cancer therapy

## Abstract

The regulation of the immune microenvironment within solid tumors has received increasing attention with the development and clinical success of immune checkpoint blockade therapies, such as those that target the PD-1/PD-L1 axis. The metabolic microenvironment within solid tumors has proven to be an important regulator of both the natural suppression of immune cell functionality and the de novo or acquired resistance to immunotherapy. Enzymatic proteins that generate immunosuppressive metabolites like adenosine are thus attractive targets to couple with immunotherapies to improve clinical efficacy. CD38 is one such enzyme. While the role of CD38 in hematological malignancies has been extensively studied, the impact of CD38 expression within solid tumors is largely unknown, though most current data indicate an immunosuppressive role for CD38. However, CD38 is far from a simple enzyme, and there are several remaining questions that require further study. To effectively treat solid tumors, we must learn as much about this multifaceted protein as possible—i.e., which infiltrating immune cell types express CD38 for functional activities, the most effective CD38 inhibitor(s) to employ, and the influence of other similarly functioning enzymes that may also contribute towards an immunosuppressive microenvironment. Gathering knowledge such as this will allow for intelligent targeting of CD38, the reinvigoration of immune functionality and, ultimately, tumor elimination.

## 1. Introduction

The burgeoning field of immuno-oncology has revealed the intricate complexities regulating tumor elimination versus tumor escape from immune detection and death, and the dysregulation that ultimately tips the scales towards escape. The clinical successes of blocking antibodies that target the braking mechanisms employed by tumors have established the use of immunotherapy as a powerful therapeutic tool to improve patient survival. However, the currently approved drugs targeting the immunosuppressive PD-1/PD-L1 or CTLA-4 axes, while efficacious in some [1,2], do not adequately address the realm of alterations that occur in tumors or the local microenvironment to suppress an anti-tumor immune response [3,4].

Emerging as a relatively new immune checkpoint is the production and accumulation of immunosuppressive metabolites in the tumor microenvironment (TME), with adenosine as a prime example. The enzymes CD39 and CD73 function in tandem to hydrolyze adenosine triphosphate (ATP) into the immunosuppressive metabolite adenosine [5]. This cascade of hydrolyzing steps ultimately acts as a shift from a pro-inflammatory response to an anti-inflammatory response, with detrimental effects towards cytotoxic CD8+ T cells, NK cells and dendritic cells, among other alterations [6,7,8]. CD38 is another well characterized ectoenzyme, with multiple functions as both an enzymatic protein as well as a receptor expressed on the cell surface [9]. Using nicotinamide adenine dinucleotide (NAD+) as a substrate, the enzymatic activity of CD38 includes the production of adenosine diphosphate ribose (ADPR) or cyclic ADPR (cADPR) [9]. Interestingly, ADPR can feed into the adenosine production pathway, providing a secondary pathway to create extracellular adenosine that bypasses CD39. Together, the myriad functions of CD38 in the microenvironment ultimately decrease extracellular NAD+, alter calcium signaling cascades, and produce immunosuppressive adenosine.

CD38 was originally identified as a lymphocyte activation marker [10,11], but our knowledge about CD38 has since evolved [12,13]. It is almost ubiquitously expressed on multiple immune populations, including T cells, NK cells, and dendritic cells, and a whole body CD38 knockout (KO) mouse demonstrates defects in dendritic cell and neutrophil migration, insufficient T cell priming and diminished humoral immunity [14,15]. CD38 has been extensively studied for its role in hematological malignancies, including chronic lymphocytic leukemia [16,17] and multiple myeloma [17,18,19]. Research on CD38 and its involvement in chronic inflammatory diseases, such as rheumatoid arthritis [20,21] and asthma [22,23], indicates that the aberrant expression and hyperactivity of CD38 can tip immune responses towards disease pathology. The understanding of how this immune cell marker may influence the progression and immune evasion within solid tumors is a relatively new field. In solid tumors, the data largely indicate an immunosuppressive role for CD38 [24,25,26], indicating the potential to utilize CD38 inhibitors in these tumors. However, the implementation of a CD38-targeting strategy in solid tumors would likely be more complicated than it may first appear. Far from inhibiting a simple enzymatic reaction, CD38 inhibition would likely have unforeseen effects, as it is a highly complex molecule capable of numerous functions. Additional research is required in order for the rational and efficacious delivery of these inhibitors, either alone or in combination with other immunotherapeutic agents, to fully realize their potential.

The focus of this review will be on the role of CD38 in hyper-inflammatory and chronic diseases in the lung such as airway hyper-responsiveness and asthma, as well as how these findings relate to the breadth of research on CD38 functioning within solid tumors including melanoma and lung cancer. CD38 is perched at a critical tipping point, often shifting the balance towards aberrant immune activity and disease progression through the alteration of the metabolic profile within tissues. The work described herein indicate the need to further explore the expression and activity of CD38—not only within immune populations but also within immunosuppressive cancer cells—to better understand the impact of CD38 on the metabolic profile within tumors and how this, in turn, influences anti-tumor immunity.

## 2. Functions of CD38

The multifunctional properties of CD38 have been previously reviewed extensively [9,12,13,27] and in this issue. Briefly, these functions are described here, but the cited literature can be reviewed for greater details about these CD38-regulated processes.

### 2.1. Enzymatic Activity

**Figure 1:**
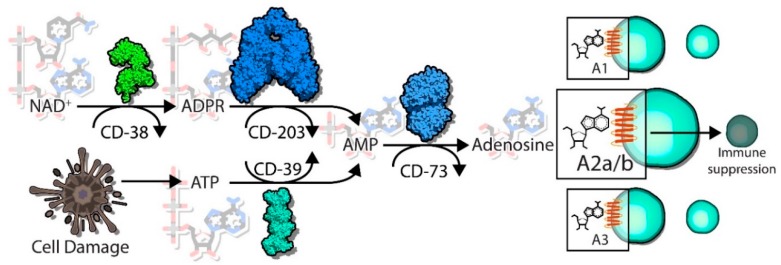
CD38 catalyzes the first step towards adenosine formation from nicotinamide adenine dinucleotide (NAD+). NAD+ present within the tumor microenvironment is catalyzed into adenosine diphosphate ribose (ADPR)—or cyclic ADPR (cADPR), not shown—by CD38. This is the first step in an alternative adenosine generating pathway, with the canonical pathway involving CD39 catalyzing adenosine triphosphate (ATP) to adenosine monophosphate (AMP). AMP is then generated from ADPR by CD203. Both pathways rely on CD73 to convert AMP into the final product adenosine, which can then promote immunosuppression in T cells via adenosine receptor 2A or 2B signaling cascades.

As outlined, the enzymatic activities of CD38 are complex. One of its known functions is as a NADase enzyme, causing hydrolysis of NAD and creating the byproducts ADPR and nicotinamide (Figure 1) [9,12,13]. CD38 can also catalyze the cyclization of NAD to produce cADPR through its ADP ribose cyclase activity, though this function may be secondary to its function as a NADase. Another known substrate of CD38 is nicotinamide adenine dinucleotide phosphate (NADP), which can be hydrolyzed into nicotinic acid adenine dinucleotide phosphate (NAADP) via a base-exchange reaction [28]. However, the functional relevance of this particular catalytic function of CD38 has yet to be fully elucidated. The requirements of an acidic pH and millimolar concentrations of nicotinic acid (NA) suggest that these reactions may only occur in vitro but not within a whole cell environment—or may only be limited to certain circumstances with high levels of NA and an acidic environment [29]. Additionally, there is also evidence that the concentration of NAADP within cells is unchanged by alteration of CD38 expression [29], suggesting CD38 may not be the main enzyme responsible for creating the NAADP byproduct.

### 2.2. Receptor/Ligand Activity

Studies utilizing agonistic antibodies revealed potential receptor functions for CD38 [30]. The administration of these antibodies altered intracellular calcium levels (though later studies appeared to decouple the enzymatic and receptor functions), as well as effected cellular proliferation. Utilization of a CD38 blocking antibody provided the first evidence for a cell-bound ligand, which was determined to be the endothelial cell marker CD31 (PECAM-1) [31,32]. These studies demonstrated that the interaction between CD38 and CD31 was similar to those seen with selectins, producing weak and dynamic interactions between immune cells and endothelial cells to promote immune cell migratory behavior. Structural studies revealed CD38 to have a non-canonical receptorial structure, with a fairly short cytoplasmic tail [33], suggesting that CD38 is unable to activate downstream signaling cascades. Thus, CD38 requires direct association with other signaling receptors in order to exert its own independent functions.

In T cells, CD38 ligation with either agonistic antibodies or with its ligand CD31 elicits downstream effects that partially overlap with T cell receptor (TCR)/CD3 activation. Specifically, the downstream cytoplasmic events following CD38 ligation include phosphorylation of PLC-γ, ZAP-70, Shc, and c-Cbl, as well as MAPK pathway activation such as Erk2 kinase activity [34]. CD38 ligation induced the expression of multiple cytokines including IL-1β, IL-6, IL-10, and IFN-γ in both T cells and monocytes [35,36]. Additional efforts determined that the TCR/CD3 complex itself is rapidly modulated following ligation of CD38 with agonistic antibodies and these molecules are thus closely associated with each other [37]. CD38 cooperates with TCR signaling cascades as a part of a supramolecular complex often found most effective within lipid rafts [38,39]. Similar studies have been completed in B cells and NK cells [40]. NK cells express high levels of CD38 [41], but despite its high expression, downstream signaling regulated by CD38 is dependent on the expression of CD16 [42], and ultimately leads to cytotoxic triggering events such as degranulation and upregulation of cytokines such as IFN-γ [43,44]. In B cells, signaling through CD38 requires B cell receptor (BCR) expression, and downstream effects differ based on differentiation stage. For example, CD38 activation in mature splenic B cells promotes their proliferation; whereas, in immature B cells within the bone marrow, CD38 can actually promote apoptosis [45]. Studies in myeloid cells demonstrated that ligation of CD38 mediates the phosphorylation of c-Cbl and subsequent association with phosphatidylinositol (PI)-3-K [46,47], leading to regulation of superoxide production [48]. Lastly, CD38 also acts as a surface molecule on dendritic cells with its expression associated with the signaling molecule CD83, thus mediating the activation of nearby T cells. Additionally, other dendritic cell migratory molecules like CD11b and CCR7 can also associate with CD38 and can regulate dendritic cell chemotaxis and trans-endothelial migration [49].

It is important to note here that the breadth of the work on CD38 and its function as a receptor has revealed disparities between human and murine CD38 in terms of tissue distribution and signal transduction [9]. Thus, any conclusions derived from murine CD38 need to be confirmed in a human model in order to be translatable to human CD38 and its role in disease progression. Additionally, there is little evidence on whether CD38 functions similarly on cells of epithelial origin, as most research efforts have focused on immune cell CD38. Thus, CD38+ solid tumors may illustrate differential phenotypes or downstream signaling cascades than those outlined for immune cells, and thus represent an additional area of research that requires expansion.

## 3. CD38 in Hyper-Inflammatory Responses

CD38 expression can push ‘normal’ inflammatory responses into hyperinflammation and aberrant pathology. In the lung, the link between CD38 and airway hyperresponsiveness (AHR) in allergic inflammation and asthma has been made evident. Specifically, CD38 activity in airway smooth muscle (ASM) cells regulates calcium signaling via the secondary messenger cADPR. However, increased CD38 expression occurs as a function of allergic inflammation, namely through an increase in pro-inflammatory cytokines such as TNF-α and IL-13. This aberrant CD38 expression alters the calcium homeostasis in ASM cells, leading to hypercontractility, proliferation and AHR [50,51,52]. CD38 knockout (KO) mice were utilized to demonstrate that whole body depletion of CD38 diminished the severity of AHR in response to an allergen or methacholine challenge, though this does not account for the influence of CD38+ immune cells in the lung parenchyma that could further influence or modulate allergic inflammation. To address this possibility, the authors performed studies utilizing bone marrow chimeras, implanting WT bone marrow into CD38-null mice [51]. These studies showed that CD38 KO mice, regardless if WT or CD38 KO bone marrow was reconstituted in the mice, have diminished airway hyperreactivity, suggesting that CD38 expression in the lung tissues contributes to AHR.

Evidence also exists for the role of CD38 in tipping the inflammatory signature towards hyperinflammation and lung disease as a result of respiratory syncytial virus (RSV) infection. RSV infection can lead to aberrant lung pathology including bronchitis. Inhibition of either CD38 directly—or the secondary messenger cADPR—reduced RSV-induced IFNs (type I/III), along with causing a reduction of interferon stimulated genes [53]. These data suggest CD38 expression can push a normal inflammatory response towards hyperinflammation and lung pathology as a result of infection. Additionally, studies have also linked CD38+ macrophages to chronic obstructive pulmonary disease (COPD), though no mechanistic studies have yet provided clear data as to the functional role of CD38 in this chronic inflammatory condition [54].

In addition to pathology within the lung, the role of CD38 in rheumatoid arthritis (RA) and collagen-induced arthritis (CIA) has also been evaluated—both diseases demarked by chronic inflammation. While the incidence of CIA was only slightly lower in CD38 KO mice compared to control mice, the severity of disease was significantly lower with CD38 KO [21]. There was a lack of induction of invariant NKT (iNKT) cells in the spleen of CD38 KO mice, as well as reduced percentages of Th1 cells in the draining lymph nodes. CD38-null bone marrow-derived dendritic cells (BMDCs) have a decreased expression of RelB, which is an important regulator of CIA, as well as a decreased MHCII expression and repressed antigen presentation [20]. CIA mice display aberrant joint structures, whereas CD38^−/−^ CIA mice have a dramatic attenuation of joint pathology, likely through the attenuation of NF-κB signaling and a decrease in proinflammatory cytokines such as IL-1β. Thus, CD38 deficiency is beneficial for the onset and/or pathogenesis of CIA.

The studies from hyperinflammatory and chronic diseases like those described above reveal that CD38 can act as a tipping point towards aberrant inflammatory response and disease progression. Additionally, they highlight that CD38 functions are complex and present on a multitude of subtypes—particularly in the lung, where airway smooth muscles cells, as well as resident immune cells, utilize CD38 in order to regulate inflammation and tissue metabolic profile. This indicates that therapeutic strategies attempting to target this molecule may require more extensive research to definitively identify the impact of treatment on all cell types with CD38 expression.

## 4. The Role of CD38 in Tumors

The interest in CD38 as a tumor-promoting molecule has grown as more research expands the field, originating in hematological diseases such as chronic lymphocytic leukemia (CLL) and multiple myeloma, but now blossoming into multiple types of solid tumors. The role of CD38 in CLL and multiple myeloma has been reviewed previously and is highlighted by other articles in this issue. As such, only a brief overview will be given here. In contrast, the influence of CD38 in driving tumor progression in solid tumors is a relatively new finding, and thus deserves review in order to determine in which direction and what further work is required in order to properly understand CD38 on tumor cells and infiltrating immune cells, as well as properly guide the development of treatment strategies.

### 4.1. Hematological Malignancies

Chronic lymphocytic leukemia (CLL) is characterized as a slow developing disease caused by the clonal accumulation of CD5+ B lymphocytes in the bone marrow, which eventually extends into the blood and secondary lymphoid organs. Due to the heterogeneous clinical outcomes for patients with CLL, early research efforts focused on identifying biomarkers or classifiers to accurately predict higher risk patients. From these studies, CD38 emerged as a dependable marker in CLL for poor prognosis, with its expression significantly correlated with a more aggressive clinical behavior [55,56]. Indeed, CLL patients with greater than 30% CD38+ B cells required more rigorous chemotherapy treatment regimens and a shorter time to first treatment [57], as well as displaying significantly shorter survival times, all suggesting a more aggressive disease course with clonal expression of CD38. Additional work has directly compared CD38+ to CD38- CLL clones and found that CD38+ cells have an enhanced migratory ability in response to the chemokine CXCL12 [58], thus enhancing their homing to lymphoid tissue, as well as higher levels of VEGF and Mcl-1 [59], indicating CD38+ CLL cells have a heightened survival advantage within the microenvironment compared to CD38- clones. Other features of CD38+ CLL cells (i.e., variable telomere lengths [60], development of new DNA mutations [61] and copy number changes [62], increased proliferation [63], etc.) all indicate a hyperproliferative phenotype of CD38+ CLL [17].

Multiple myeloma (MM) is a hematological cancer type arising from the clonal expansion of malignant plasma cells in the bone marrow. CD38 expression was discovered to be uniformly high on MM cells within the bone marrow niche [64,65], and its expression was relatively lower on osteoblasts and mature osteoclasts. Daratumumab, a human anti-CD38 antibody, effectively induces myeloma cell death mostly through antibody-dependent cell-mediated cytotoxicity (ADCC), but can also elicit complement-dependent cytotoxicity(CDC) and antibody-dependent cellular phagocytosis (ADCP) [41,66], leading to its FDA-approval for treatment of treatment refractory multiple myeloma in 2015 [67]. Additional studies demonstrated that treatment with daratumumab not only directly kills myeloma cells but also causes the depletion of CD38+ immunosuppressive cells like myeloid derived suppressor cells (MDSCs) and Tregs, relieving local immunosuppression and improving the cytotoxic CD8+ immune response [68].

Despite the clinical success of daratumumab and its minimal cytotoxicity profile, patient responses were variable and most eventually develop progressive disease when delivered as a monotherapy. One likely cause of acquired resistance is the downregulation of CD38 expression in both bone marrow and circulating myeloma cells after a single daratumumab infusion [69,70]; thus, the use of anti-CD38 therapy in multiple myeloma patients has revealed that these antibodies may be most efficacious when delivered as part of a combination treatment strategy. Indeed, the FDA recently approved combination daratumumab with lenalidomide for the first line treatment of multiple myeloma in newly diagnosed patients who are ineligible for autologous stem cell transplantation [71]. An additional mechanism of resistance likely stems from the depletion of CD38+ cytotoxic immune cells, specifically NK cells [70]. The primary mechanism of action for daratumumab is ADCC, which is largely mediated by the action of NK cells. Elimination of this cell population occurs via CD38+ NK cell apoptosis [72] and, in turn, diminishes daratumumab efficacy [73] and also can increase the risk of infectious complications [74]. Knowledge about CD38-targeting strategies and mechanisms of resistance from these studies in myeloma may prove useful as researchers in the solid tumor field attempt to apply anti-CD38 therapeutic strategies especially for tumor types which have shown to have aberrant and dynamic CD38 activity, which will be described in the next section.

### 4.2. Solid Tumors

The understanding of the role CD38 plays in the progression and immune evasion of solid tumors is only just beginning. Though limited, there have been studies in melanoma, glioma, esophageal, cervical, and lung cancers. In these diseases, CD38 appears to function as a tumor-promoting factor, though conflicting data does exist for the influence of CD38 in the progression of prostate cancer. While CD38 expression on cells within the TME, such as T cells, myeloid cells or macrophages, still exists as the prime focus of these research efforts, more data is beginning to accumulate regarding the influence of CD38 expression on the tumor cells.

In esophageal cancer, CD38 was found to be upregulated on myeloid-derived suppressor cells (MDSCs), occurring through the accumulation of tumor-derived secreted factors including IL-6, IGFBP3, and CXCL16 [26]. These CD38+ MDSCs were characterized as being more immature in their differentiation status than those with low expression of CD38, thus having a greater ability to suppress the activity of T cells within tumors. Additionally, anti-CD38 antibody treatment experiments revealed that the expression of CD38 on MDSCs is functional in promoting tumor growth, as implantation of MDSCs crosslinked with an anti-CD38 antibody significantly repressed tumor growth compared to CD38^high^ MDSC implantation [26]. These studies provide insight into the role of CD38+ MDSCs in the TME of esophageal tumors and is supported by a similar study in advanced colorectal cancer patients [75]. While providing evidence to support the use of anti-CD38 therapy in these solid tumor models, the expression of CD38 on other immune populations or even the tumor cells themselves was not analyzed in these studies.

Similar studies completed in glioma transplantation models of cancer have found that CD38 null mice have reduced glioma expansion and extended life spans as compared to glioma bearing wildtype mice [25]. Analysis of subpopulations of tumor-associated microglia/macrophages (TMM) revealed that F4/80 macrophages were found to be significantly reduced in glioma-bearing CD38 KO mice. Tumor cell death was significantly higher in CD38 KO mice [25], and follow-up studies demonstrated that similar results can be obtained with pharmacological targeting of CD38 through the administration of the inhibitor K-rhein [76], which is a flavonoid frequently used as pharmacologic inhibitor of CD38 [22]. These studies reveal an additional mechanism by which CD38 expression in the TME—perhaps through recruitment or survival of tumor-promoting macrophages—favors tumor growth.

Research on the influence of CD38 in lung cancer has focused on tumor-expressing CD38 and how it impacts disease progression. Knockout of CD38 in human A549 cells reduced the invasive and clonogenic abilities, as well as significantly reducing tumor growth in a xenograft model of lung cancer [77]. As this study was completed in nude mice, this reduction in tumor growth is likely due to CD38 on tumor cells and does not account for the impact of CD38-expressing immune cells in promoting tumor growth. Lastly, this study demonstrated CD38 protein and RNA upregulation in a percentage of human lung cancer specimens [77]. Similar work was completed in cervical cancer, which found CD38 RNA and protein expression to be high in cancerous tissue in the cervix when compared to normal tissue [78]. Follow-up studies from this group determined a distinct growth advantage of cervical cancer cells in in vitro proliferative analyses, as well as in a nude mouse model of cervical tumor growth [79]. The likely mechanism for the regulation of cell proliferation and survival is the prevention of mitochondrial apoptosis through a lack of intracellular calcium stores with CD38 overexpression, as well as downregulation of p53 signaling.

Studies from our group have added to these data by demonstrating tumor-expressing CD38 is significantly upregulated in Kras/p53 mutant lung tumors during treatment with anti-PD-L1 or –PD1 antibodies, and this upregulation promotes treatment resistance [24]. Genetic and proteomic analyses revealed that CD38 is upregulated in response to a re-invigorated immune response resulting from treatment with PD(L)-1 blocking antibodies, namely through all-trans retinoic acid (ATRA) and a type I interferon response within the tumor. The murine evidence was supported with human lung tumor data, in which ~25% of lung tumor cells expressed CD38 in strong correlation to the degree of T cell infiltration, while in melanoma patient samples, CD38 is upregulated after treatment with anti-PD-1. In multiple murine models, genetic manipulation of CD38 on the tumor cells, or treatment with either small molecule inhibitors or an anti-CD38 antibody, regulated tumor growth in a CD8 T cell-dependent fashion and produced a broader anti-tumor immune profile. The upregulation of CD38 promotes an increase in adenosine within the TME, and the subsequent repression of the cytotoxic T cell response. Pharmacological targeting of the adenosine pathway via adenosine receptor inhibition was able to reverse the immunosuppression caused by CD38 upregulation [24]. These data from our laboratory provide strong rationale for the use of CD38 inhibition in conjunction with immune checkpoint inhibitors in order to combat acquired resistance, and indeed have found that combinations of blocking antibodies towards CD38 and PD-(L)1 provide a robust response in preclinical models of lung cancer.

The researches completed in solid tumors are largely supportive of each other, in that regardless of the cell type expressing CD38, the effect is immunosuppressive and tumor-promoting. However, the data generated from studies on the influence of CD38 in prostate cancer are disparate from these findings. Low expression of CD38 enriches for luminal progenitor cells in human prostate. CD38 low luminal cells are localized in proximity to prostatic inflammation, and low CD38 is prognostic for biochemical recurrence and metastasis [80]. CD38 mRNA was reduced in metastatic, castration-resistant prostate cancer compared to localized prostate cancer, and protein expression was inversely correlated with recurrence. Overexpression of CD38 in PC depleted extracellular, but not intracellular, NAD+ levels, though the functional relevance of this depletion of extracellular NAD+ was not elucidated in these studies [81]. Another study confirmed that CD38 expression is inversely correlated with tumor progression in prostate cancer, and this corresponds with increased NAD+ levels in the tumor [82]. The overexpression of CD38 in prostate cancer cells decreased proliferation with an increase in cell doubling time, as well as decreased glycolytic and metabolic capacity. Lastly, these studies demonstrated that CD38 activity increases pAMPK with subsequent inhibition of fatty acid and lipid synthesis [82], so loss of CD38 with tumor progression correlates with decreased pAMPK and increased fatty acid/lipid synthesis. As prostate cancer has a lipogenic phenotype, the lack of fatty acid and lipid synthesis as a result of CD38 expression would likely be detrimental for successful tumorigenesis. Thus, the differential metabolic needs of prostate cancer, as compared to other solid tumors described above, may hint to the varying impact of the expression of CD38 in these diseases.

Taken together, these studies indicate that therapeutic strategies that target CD38 activity would likely be effective in solid tumors in addition to hematologic malignancies like multiple myeloma. The exception to this may exist for those tumor types which require increased NAD+ pools for metabolic needs like fatty acid and lipid synthesis, as described in prostate cancer. Of note, the aforementioned research did not assay the expression of CD157, a CD38 homolog, or other ectoenzymes that regulate metabolite levels in the extracellular environment that may be immunosuppressive.

## 5. Adenosine Generating Pathways

### 5.1. CD39/CD73

Adenosine triphosphate (ATP) is released from dying tumor cells as a result of the generally hypoxic and nutrient-poor microenvironment that occurs with increasing tumor progression (Figure 1). ATP is a pro-inflammatory metabolite, recruiting neutrophils cells to a site of damage (i.e., a tumor) as one of the first lines of immune defense, as well as playing a role in chemotaxis and activation of macrophages and dendritic cells [83]. The presence of ATP, while pro-inflammatory, can lead to the increased expression of ectoenzymes which can hydrolyze metabolites, notably CD39 (ecto-nucleoside triphosphate diphosphohydrolase 1, E-NTPDase1). Through two steps of phosphohydrolysis, CD39 creates adenosine diphosphate (ADP), and—in a less efficient step—adenosine monophosphate (AMP). While the generation of AMP itself is not considered to be immunosuppressive, the final dephosphorylation reaction catalyzed by the enzyme CD73 (ecto-5’-nucleotidase, Ecto5’NTase) generates adenosine from AMP, ultimately switching the microenvironment towards immunosuppression. The accumulated adenosine within the TME can then regulate neighboring immune cells expressing the P1 purine adenosine receptors (ADORAs). The expression and activity of ADORA2A and ADORA2B (A2A and A2B, respectively) on immune cells leads to activation of intracellular signaling cascades such as those regulated by cyclic AMP (cAMP)/PKA, which ultimately negatively regulate immune functions via NF-κB and JAK/STAT signaling pathways. Immune cells that are inhibited by extracellular adenosine via the activity of A2A and A2B are cytotoxic T cells, NK cells, and dendritic cells, whereas macrophage polarization can also be impacted through the actions of adenosine [6]. Together, the pro-inflammatory signature of ATP is shifted towards anti-inflammatory effects via the coordinated efforts of CD39 and CD73 in the canonical pathway.

### 5.2. CD38/CD203a/CD73

As mentioned above, one of the enzymatic byproducts generated by the metabolism of NAD+ by CD38 is ADPR, either directly or indirectly through cADPR generation. The further breakdown of extracellular ADPR to AMP can be regulated by CD203a (ecto-nucleotide pyrophosphatase/phosphodiesterase 1, NPP1) [84,85] (Figure 1). Once AMP is generated, this pathway is still dependent upon CD73 to convert the final step of AMP to adenosine, providing some overlap between the canonical and noncanonical adenosine generating pathways. Interestingly, cells that co-express CD38 and CD203a are able to generate high levels of AMP through ADPR, though the discontinuous expression of CD73 (meaning expression on a different cell type) may be sufficient for the completion of the final step of this adenosine-producing pathway [86].

## 6. Immunosuppressive Functions of Adenosine-Producing Pathways

Accumulation of adenosine has been repeatedly shown to occur in tumors and is immunosuppressive in its impact on the intratumoral immune populations. Thus, analysis of the upstream enzymes, especially CD39 and CD73, have been warranted to better understand the processes which drive this immunosuppression.

The involvement of CD39 and CD73 in autoimmune diseases reveals that these enzymes can disrupt the natural balance of inflammatory signals, similar to what has been observed for CD38. Namely, the CD39/CD73 pathway may be involved in the onset of multiple sclerosis through a mouse model termed experimental allergic encephalomyelitis (EAE). The knockout of CD73 within these mice significantly reduced the development of EAE, with fewer pathogenic T cells infiltrated into the central nervous system [87]. However, a conflicting study found that IFN-beta treatment, commonly utilized as a treatment for MS patients, can actually increase the expression of CD73 and levels of adenosine [88]; thus, further investigations are required to definitively illuminate the role of CD39/CD73 in MS development and progression. Additionally, CD39 has been determined to be important in the progression of HIV/AIDS. CD4+ FoxP3+ T regulatory cells in HIV-1 positive patients express high levels of CD39 [89]. The expansion of this population of CD39+ Tregs correlates with decreased CD4+ T counts in HIV-1 patients, and patients with a gene polymorphism in CD39 corresponding with lower expression have a slower progression to AIDS. These data provide evidence for both CD39 and CD73 in contributing to chronic immune-related diseases.

In tumors, CD73 and CD39 were found to both be overexpressed and functionally active in producing adenosine in ovarian cancer cells [90], and melanoma cells expressed the enzymes for both the canonical (CD39/CD73) and alternative (CD38/CD203a) pathways for adenosine production [91]. Similarly, CD73 correlates with poor prognosis in triple negative breast cancer and high grade serous ovarian cancers [92,93]. CD73 expression in breast cancer contributes to tumor growth and metastasis in immunocompetent mice, which could be reversed by blocking CD73 or the adenosine receptor expressed on the 4T1 breast cancer cells. Together, the data indicate an immunosuppressive role of tumor-expressing CD73 and the extracellular adenosine that results from its overexpression [94]. These data were supported by CD73 knockout mice which showed similar decreases in tumor growth and metastasis, effects which stemmed from both hematopoietic and nonhematopoietic cell expression of CD73 [95]. CD73 expression is heterogeneous in melanoma, though it was significantly associated with p53 mutations and trended with Braf mutations [96]. Inhibiting the adenosine receptor A2A—especially in the immune compartment—was able to reduce tumor initiation in a Braf/Pten mouse model, whereas direct adenosine stimulation promotes metastatic potential and outgrowth with no impact on primary tumor growth. These data suggest that CD73 and downstream adenosine accumulation are important for later stage tumors to metastasize and evade immune elimination during metastasis [96]. Despite these data and there being a significant increase in expression with disease stage, CD73 score was not found to be an independent prognostic factor in melanoma [96]; however, clinical data from NSCLC patients revealed CD73 as an independent indicator of poor prognosis in overall survival in those harboring EGFR or ALK mutations [97,98]. These data suggest that certain solid tumors may be more dependent on adenosine accumulation for the evasion of anti-tumor immunity, or that other pathways such as CD38/CD203a may need to be considered as well.

CD73-expressing tumor cells suppress T cell proliferation, which was abrogated when cultured with A2A blocking antibodies, suggesting that adenosine generated by CD73-expressing cells leads to the suppression of T cell responses [99]. Knockdown of CD73 on ovarian tumor cells was also able to reinvigorate T cell proliferation. CD73-derived adenosine from tumor cells was also shown to significantly promote the apoptosis of T cells, which again could be rescued through inhibition of A2A signaling. These data were corroborated in vivo, with anti-tumor T cell activation and effector functions improving with the depletion of CD73, likely through diminishing intratumoral adenosine concentrations—thus allowing for proper T cell activation and cytolytic functions [99].

## 7. Targeting Adenosine-Generating Pathways

Due to the emergence of these adenosine-generating enzymes as potential immune modulatory pathways that can be targeted, extensive efforts in demonstrating the efficacy of these agents in vitro and in preclinical models have been underway. For example, there is success of co-targeting CD73 and CD39 in melanoma and fibrosarcoma [100], reinvigorating anti-tumor immunity alone or in combination with oxaliplatin. Additionally, combinations with currently approved immunotherapies have been extensively investigated, finding there to be an increase in efficacy of PD-1 blockade when given in conjunction with anti-CD73 inhibitors [101]. Similar effects were observed in combining anti-PD-1 with the blockade of the downstream adenosine receptor A2A [102]. Interestingly, targeting the CD39/CD73/ADORA axis may have non-redundant functions, in that co-targeting CD73 and the A2A receptor improves the efficacy of either inhibitor alone [103], though this study was not completed in the context of improving the efficacy of currently FDA-approved immunotherapy antibodies such as those that block PD(L)1. This preclinical work has culminated in the initiation of clinical trials which combine each of these agents with PD-1/PD-L1 blocking antibodies [104,105] (ClinicalTrials.gov identifiers: NCT02503774, NCT03549000, NCT03454451, NCT02403193, NCT02655822, NCT03207867). Similar to these efforts to target the canonical pathway via CD39/CD73 inhibitors, data generated from our laboratory reveal similar efficacy may be achieved by targeting CD38. We demonstrated that the expression of CD38 on lung cancer cells is a novel avenue of acquired resistance to single agent PD-1 checkpoint inhibition [24]. Both in vitro T cell cytotoxicity assays, as well as in vivo combination treatment regimens, suggest that the upregulation of CD38 leads to an immunosuppressed TME and targeting CD38 together with the blockade of the PD-1 axis leads to improved anti-tumor immune responses.

Like the CD73/A2A treatment strategies being currently pursued in the clinical setting, targeting CD38 would similarly limit microenvironmental adenosine. As described earlier, this would likely promote tumor elimination, as high levels of adenosine trigger immunosuppression. However, targeting the enzymatic ability of CD38 would also more generally disrupt NAD processing and calcium signaling. Increased NAD+ is a danger signal for immune response, much like extracellular ATP [83]. NAD+ has a longer half-life in tissues when compared to ATP, as well as the ability to access draining lymph nodes, so the accumulation of NAD+ metabolites may have far reaching impacts on antitumor immunity. CD38^−/−^ mice demonstrate an increased half-life of NAD+ in biological fluids [106], thus suggesting that the inhibition of CD38 via specific blocking antibodies or enzymatic inhibitors would have a similar influence on intratumoral NAD+ accumulation. Activation of the receptor P2Y11R, which responds to higher extracellular concentrations of NAD+, on granulocytes increases chemotaxis and the production of superoxide [107]. NAD+ can also serve to recruit and activate neutrophils [108,109]. The expression of P2X7R receptor in conjunction with ART2 on the cell surface of T cells results in cell death in response to extracellular NAD+; however, activated T cells appear to be resistant to this process, as they downregulate the expression of surface ART2, which is the necessary step for modification of P2X7R and subsequent cell death signaling. Thus, while extracellular NAD+ could promote T cell death within tumors, in likelihood, the activated cytotoxic T cells would be resistant and still function to mediate tumor cell death [110,111].

Conversely, CD4+ Tregs are more sensitive to NAD/ATP-induced cell death when compared to normal T cells [112]. Blocking CD38 in vivo in a mouse model of lung cancer led to a significant reduction of the total Treg cell population within the TME [24], potentially due to expression of CD38 on these cells and/or occurring through NAD+ accumulation and Treg cell death. Overall, a more effective anti-tumor response occurred with blockade of CD38 in this model of lung cancer, and similar effects have been shown though direct NAD+ injection [112]. These data support the argument that CD38 targeting may be a reasonable treatment strategy to explore outside the field of hematological malignancies, such as for the treatment of solid tumors like lung cancer.

## 8. Remaining Questions Surrounding CD38 in Solid Tumors

**Figure 2:**
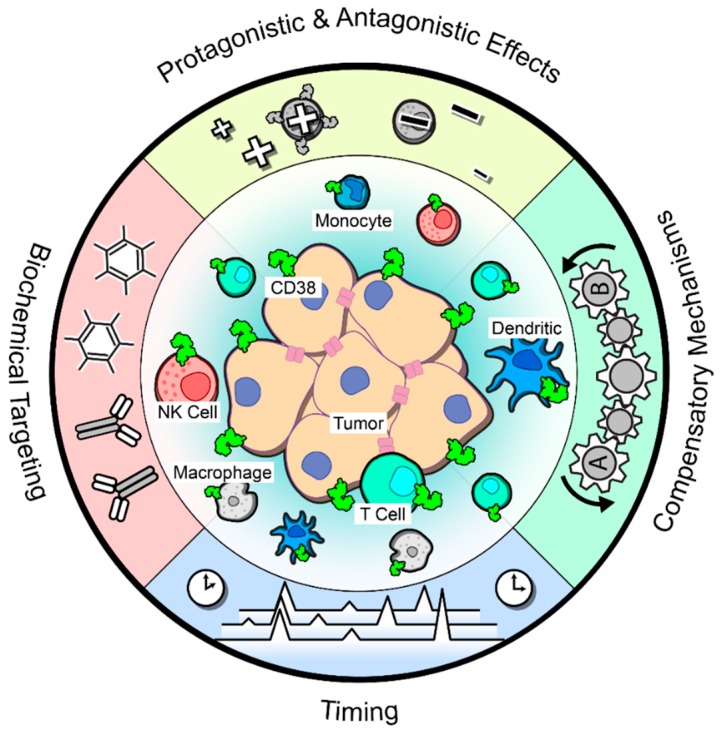
The expression of CD38 within a complex solid tumor microenvironment. The schematic highlights the diversity of CD38 expression within solid tumors, including on the tumor cells as well as resident and infiltrating immune cell populations. As it stands, there are several areas of research necessitating further studies for better understanding of the impact of CD38 targeting in these tumors. Because CD38 functions on T cells, natural killer (NK) cells, and dendritic cells, anti-CD38 treatment may actually prevent the activity of these anti-tumor immune cells and thus decrease efficacy of this treatment strategy (yellow). Additionally, there are a multitude of ways to effectively target CD38, either through blocking antibodies with or without enzymatic inhibition or via small molecule inhibitors which inhibit NADase activity of CD38 (red). Which treatment strategy would be the most efficacious in solid tumors remains a major unknown. Another complicating factor is the potential for compensatory mechanisms which could overcome CD38 inhibition, such as the canonical adenosine generating pathway utilizing CD39 and CD73 (green). Lastly, CD38 likely requires a combinatory treatment strategy in solid tumors to prevent resistance and improve efficacy; thus, what agents to combine with CD38-targeting agents and the timing of these combinations requires future work to best utilize these agents in the treatment of solid tumors (blue).

Together, these aforementioned studies suggest that blocking CD38 in solid tumors would reduce the anti-inflammatory signature and thus reinvigorate an anti-tumor T cell response. Despite this logic and the supporting pre-clinical data, a phase Ib/II clinical trial combining daratumumab with the PD-L1 blocking antibody atezolizumab in advanced or metastatic non-small lung cancer was recently terminated due to a lack of improved response over atezolizumab monotherapy [113]. The basis for this result is unknown and formal report of the trial and biomarker analyses are eagerly awaited. Thus, many factors remain unknown when considering targeting CD38 alone or in combination with anti-PD(L)-1 antibodies (Figure 2).

### 8.1. Pro- and Antagonistic Effects

As stated earlier, the expression of CD38 is nearly ubiquitous in immune populations, including T cells and natural killer (NK) cells. Extensive data has demonstrated the cooperation of CD38 with TCR signaling on T cells [37,38,39]; thus, the expression of CD38 and its activity in TCR signaling or cytotoxic functioning on tumor-infiltrating T cells needs to be more adequately investigated to determine whether disrupting CD38 via pharmacological inhibition would also impact T cell activation and cytotoxicity. Additionally, CD38 functions as a cytotoxic triggering molecule in activated NK cells by upregulating the release of IFN-γ and TNF-α and promoting degranulation [43]. As discussed earlier, work from multiple myeloma clearly demonstrates that NK cells play a vital role in the therapeutic efficacy of daratumumab treatment by inducing ADCC and ADCP [73]. However, a follow-up study highlighted that NK cell depletion in response to daratumumab treatment does not correlate with patient response [114], so more research is warranted in order to determine whether CD38+ NK cells are truly required for tumor elimination. Besides T cells and NK cells, CD38 is also expressed on dendritic cells and can induce a Th1 response [115,116], again highlighting a potentially anti-tumor immune population that may be inhibited with CD38 targeting and complicate treatment efficacy. These data indicate the need to identify potential on-target but off-tumor effects of anti-CD38 agents that may impair effective tumor cell elimination.

### 8.2. Biochemical Targeting

In addition to the complexity of CD38 expression on immune subpopulations, there are insufficient data to definitively indicate which function of CD38 would need to be targeted in order for effective tumor cell killing, or whether inhibition of both enzymatic and receptor functions would be necessary. Evidence from T cells demonstrate that these two functions of CD38 are, in fact, independent of each other [9], and thus CD38 functioning within solid tumors may vary based on the circumstances or on which population(s) of cells are expressing the molecule. Data generated from our laboratory suggested that the enzymatic function of CD38 on the tumor cells is integral to the immunosuppressive microenvironment, namely through the accumulation of adenosine and adenosine receptor activation on tumor infiltrating T cells [24]. However, the receptor function of CD38 was not fully explored in this model, nor was the expression of CD38 on immune subpopulations within the tumor.

Because of its multifunctional properties both as an ectoenzyme and as a cell-surface resident protein with receptor functions, numerous targeting strategies have been explored. These include blocking antibodies that largely do not inhibit enzymatic functions like the FDA-approved daratumumab [117] or those that also partially block enzymatic activity, such as isatuximab [117,118]. Additionally, small molecule inhibitors have been developed with varying mechanisms of action [22]. One of these is 78c, discovered from a high-throughput screen and shown to act as an “NAD boosting” agent with an improved pharmacokinetic profile over previous molecules [119,120]. In order to improve detrimental on-target effects on normal CD38-expressing cells, unique tools such as CD38-Attenukine [121] and CD38 CAR T cells [122] have been created. The CD38-Attenukine molecule fused an anti-CD38 antibody with a mutated form of IFN-α which lowers the potency of IFN-α for its receptor IFNAR. Thus, normal, CD38-negative or CD38-low cells are spared with selective killing of CD38+ cells [121]. This inventive strategy demonstrated clear efficacy in multiple models of myeloma, but may also present an opportunity for the treatment of solid tumors which rely on normal CD38-expressing immune cells for tumor cell elimination. Similarly, CD38 CAR T cells have been developed for the targeting of CD38+ multiple myeloma cells [122], but on-target/off-tumor effects limited its efficacy; thus, the development of lower potency CD38 CAR T cells has improved specific on-tumor effects [123]. Taken together, the multitude of available CD38-targeting agents that exhibit efficacy in myeloma and other CD38+ malignancies need to be studied extensively in the solid tumor field in order to identify which tools will provide the best anti-tumor response in solid tumors.

### 8.3. Compensatory Mechanisms

Sufficient data is lacking to identify whether targeting CD38 alone would effectively repress adenosine in the microenvironment, as generation of this metabolite represents one of the main ways CD38 promotes immunosuppression. Most publications in the field of adenosine generation especially in tumor biology focus on a single ectoenzyme or a single adenosine-generating pathway, but rarely take into account the interconnection of these pathways, or whether compensatory mechanisms exist able to upregulate an alternative pathway when one is blocked. This type of acquired resistance has been shown in other therapeutic targeting strategies and cannot be ruled out here. Thus, it is unclear whether targeting CD38 in conjunction with another pathway member—such as CD39, or the downstream adenosine receptor A2A or A2B—would be required in order to achieve the effective downregulation of intratumoral adenosine, and the reinvigoration of immune functionality.

### 8.4. Effects of Timing

Lastly, the timing of combination treatment strategies is another important issue to consider in order to cause the most effective tumor elimination. Recent data suggest that administration of anti-PD(L)-1 treatment prior to proper T cell priming leads to immunotherapy resistance through the upregulation of PD1+CD38^high^ T cells in a melanoma tumor model [124]. This could be overcome by pretreatment with vaccination in order to fully prime intratumoral CD8+ T cells, circumventing the formation of these exhausted T cells which can no longer recover their cytotoxic capabilities even in the face of anti-PD-1. These data suggest that simultaneous treatment may be an effective strategy. However, this study did not examine whether co-treatment with a CD38-blocking antibody would be efficacious in preventing the accumulation of this immunosuppressive population. This could be a possibility, as similar results were obtained with daratumumab treatment in terms of suppressing CD38+ Tregs and MDSCs in myeloma [68]. Data generated from our group revealed that simultaneous treatment of anti-PD-L1 and anti-CD38 effectively prevented lung tumor growth [24]. The authors also demonstrated the efficacy of a staggered treatment strategy, in which tumors were first treated with an anti-PD-L1 antibody until the point of resistance, and then followed up with anti-CD38 treatment. Because single agent CD38 blockade had a marginal effect on tumor growth, the treatment with anti-PD(L)-1—either first or in combination—is likely necessary for an optimal immune response.

Thus, further work diving into the complexities of CD38 on both tumor cells and immune cells, as well as the expression and activity of other ectoenzymes with similar functions, need to be completed in order to provide a greater understanding of the role CD38 within solid tumors. This knowledge will provide a solid foundation to build upon, in order to most effectively implement combination treatment strategies to rectify aberrant metabolic profiles within tumors, reinvigorate the immune response and, ultimately, improve tumor elimination.

## 9. Conclusions

CD38 has dual functions as an ectoenzyme with multiple substrates and byproducts that feed into calcium signaling and adenosine production, and as a cell-surface expressed receptor that can promote a migratory phenotype and signaling cascades such as those involved in TCR signaling. Compounding this multifunctional complexity is the nearly ubiquitous expression of CD38 on cells within the immune system, with expression found on NK cells, T cells and dendritic cells at varying levels based upon differentiation and activation status.

The role of CD38 in multiple myeloma and CLL has been studied extensively, igniting the development of numerous treatment strategies to specifically target malignant cells while attempting to spare those normal cells that rely on CD38 expression for functional activity. The impact of CD38 expression within a heterogeneous solid tumor microenvironment, and specifically its expression on the tumor cells, remains understudied. The current status of the field points to CD38 as capable of shifting the local tumor metabolic microenvironment from pro-inflammatory to anti-inflammatory, indicating CD38 as a potential mechanism of resistance to currently approved immunotherapy agents, such as PD(L)-1 blocking antibodies.

While CD38 and other metabolic regulators within the TME, such as CD73, have gained traction as novel immunomodulating molecules and, thus, as potential targets for treatment, it remains unclear how inhibition of CD38 in solid tumors may impact anti-tumor immune functionality within these same tumors. More extensive research is imperative, focusing on exactly which tumor-infiltrating immune populations express and utilize CD38, what occurs downstream of CD38 upregulation in these immune cells as well as in the tumor cells, and how best to target this complicated molecule in terms of class of inhibitor and potential combinations to improve efficacy. Without addressing at least some of these remaining unknowns, the outcome of CD38 targeting within solid tumors may fall short in a clinical setting, and thus an important opportunity would be missed. The more we can learn about this multifaceted protein and those with similar functions within a solid tumor microenvironment, the more intelligently we can inhibit its activity to reinvigorate a pro-inflammatory immune response to effectively eliminate the tumor.
